# Therapies with CCL25 require controlled release via microparticles to avoid strong inflammatory reactions

**DOI:** 10.1186/s12951-021-00830-7

**Published:** 2021-03-25

**Authors:** J. Spinnen, K. Fröhlich, N. Sinner, M. Stolk, J. Ringe, L. Shopperly, M. Sittinger, T. Dehne, M. Seifert

**Affiliations:** 1 Tissue Engineering Laboratory, BIH Center for Regenerative Therapies, Department for Rheumatology and Clinical Immunology & Berlin Institute of Health at Charité–Universitätsmedizin Berli, BCRT, Charitéplatz 1, 10117 Berlin, Germany; 2 Institute of Medical Immunology and Berlin Institute of Health Center for Regenerative Therapies, Institute of Medical Immunology, Charité–Universitaetsmedizin Berlin, corporate member of Freie Universitaet Berlin and Humboldt-Universitaet Zu Berlin, Augustenburger Platz 1, 13353 Berlin, Germany; 3 DZHK (German Center for Cardiovascular Research), partner site Berlin, Germany

## Abstract

**Background:**

Chemokine therapy with C–C motif chemokine ligand 25 (CCL25) is currently under investigation as a promising approach to treat articular cartilage degeneration. We developed a delayed release mechanism based on Poly (lactic-co-glycolic acid) (PLGA) microparticle encapsulation for intraarticular injections to ensure prolonged release of therapeutic dosages. However, CCL25 plays an important role in immune cell regulation and inflammatory processes like T-cell homing and chronic tissue inflammation. Therefore, the potential of CCL25 to activate immune cells must be assessed more thoroughly before further translation into clinical practice. The aim of this study was to evaluate the reaction of different immune cell subsets upon stimulation with different dosages of CCL25 in comparison to CCL25 released from PLGA particles.

**Results:**

Immune cell subsets were treated for up to 5 days with CCL25 and subsequently analyzed regarding their cytokine secretion, surface marker expression, polarization, and migratory behavior. The CCL25 receptor C–C chemokine receptor type 9 (CCR9) was expressed to a different extent on all immune cell subsets. Direct stimulation of peripheral blood mononuclear cells (PBMCs) with high dosages of CCL25 resulted in strong increases in the secretion of monocyte chemoattractant protein-1 (MCP-1), interleukin-8 (IL-8), interleukin-1β (IL-1β), tumor-necrosis-factor-α (TNF-α) and interferon-γ (IFN-γ), upregulation of human leukocyte antigen-DR (HLA-DR) on monocytes and CD4^+^ T-cells, as well as immune cell migration along a CCL25 gradient. Immune cell stimulation with the supernatants from CCL25 loaded PLGA microparticles caused moderate increases in MCP-1, IL-8, and IL-1β levels, but no changes in surface marker expression or migration. Both CCL25-loaded and unloaded PLGA microparticles induced an increase in IL-8 and MCP-1 release in PBMCs and macrophages, and a slight shift of the surface marker profile towards the direction of M2-macrophage polarization.

**Conclusions:**

While supernatants of CCL25 loaded PLGA microparticles did not provoke strong inflammatory reactions, direct stimulation with CCL25 shows the critical potential to induce global inflammatory activation of human leukocytes at certain concentrations. These findings underline the importance of a safe and reliable release system in a therapeutic setup. Failure of the delivery system could result in strong local and systemic inflammatory reactions that could potentially negate the benefits of chemokine therapy.

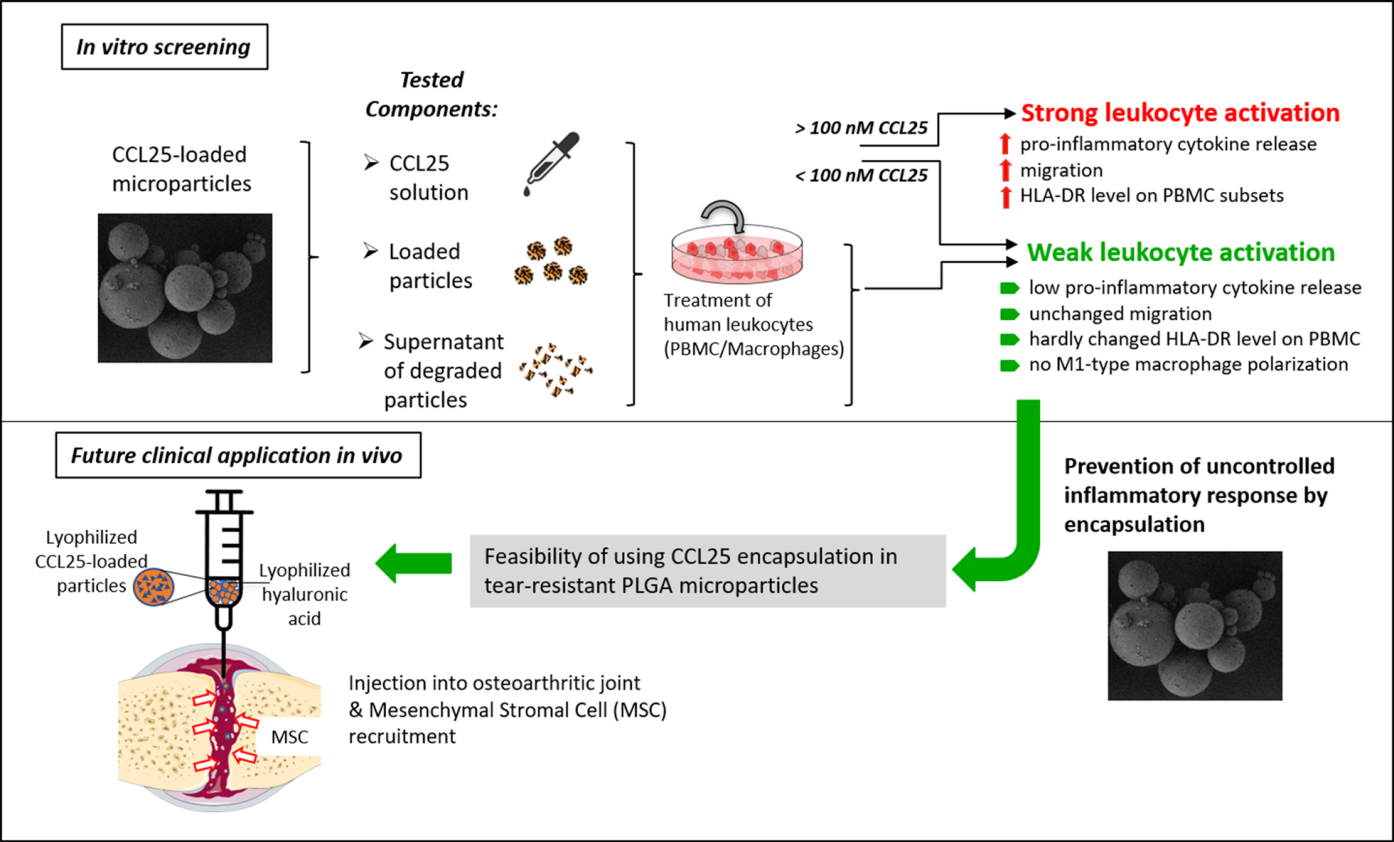

## Introduction

Research in the field of regenerative medicine is increasingly revealing the enormous potential of the human body's inherent self-healing powers. Chronic tissue damage, as seen in conditions such as osteoarthritis (OA) are not ignored by the body but mechanisms to repair damage are constantly being attempted. Unfortunately, these repair processes are often not perfectly synchronized with the underlying pathological conditions or are negatively affected by the inflammatory processes present. In OA, for example, chondrocytes in the deeper cartilage tissue respond to degeneration of the outer tissue layers with increased expression of cartilage-specific matrix proteins such as collagen II and aggrecan. However, these mechanisms are not sufficient to repair the tissue damage [1, 2].

Therefore, the tissue requires a general change in microenvironment to modulate chronic inflammation and provide pro-regenerative stimuli. Besides the injection of growth factors or instrument-based stimulation, multiple studies have shown that the intraarticular application of mesenchymal stromal cells (MSCs) can sway the microenvironment towards a pro-regenerative direction owing to their immune-regulatory and proliferation-promoting secretome [3–5]. However, the efficacy of these treatments remains very limited and the results may not justify the cost of an ex vivo cell therapy. To enhance the cost-effectiveness and efficacy of MSC targeting therapies, the application of chemokines embedded in certain release devices are of growing interest. Chemokine enriched biopolymers enable both mechanical closure of the lesion as well as delayed release of their regenerative factors [6, 7]; an approach called in-situ tissue engineering [8]. Certain chemokines have been shown to both attract certain cells like MSCs to the site of application, as well as increase the tissue producing capacities of the resident cells. So far, multiple chemokine candidates are under investigation for their suitability, with C–X–C motif chemokine ligand 12 (CXCL12) and IL-8 being among the most popular [9, 10].

In previous work, we identified C–C motif chemokine ligand 25 (CCL25) as a potent candidate for therapeutic chemoattraction of MSCs through broad-based migration assays [11, 12]. Because the knee joint is thought to contain many loculated MSCs, and the knee as a preformed body cavity is well suited for injection therapy, the efficacy of CCL25 for osteoarthritis was investigated. In an in vivo OA model (Dunkin-Hartley guinea pig), one injection per week of CCL25 dissolved in hyaluronic acid (HA) into affected joints for four consecutive weeks resulted in up to 28% less cartilage damage (graded by the histopathological OARSI Score) in the CCL25-treated joint compared with the untreated joint and HA and sodium chloride (NaCl) control groups [13, 14]. After proof of concept, a release system based on poly (lactic-co-glycolic acid) (PLGA) microparticles was developed that allows a single injection followed by controlled, sustained release of CCL25 into the joint over several weeks. Importantly, microparticles with diameters of ~ 50–100 µm allow for intraarticular injection using a syringe instead of arthroscopic implantation. On the one hand, this saves the patient a hypothetical invasive procedure, on the other hand, the PLGA degradation kinetics enable steady level of chemokines to recruit sufficient amounts of cells while avoiding potentially harmful peak concentrations that could result from multiple injections [15, 16]. PLGA was chosen because it is already successfully used as a microencapsulation material (also in the form of microparticles [17, 18]), encapsulation of proteins is well standardized and due to its biocompatibility and biodegradability is considered safe for use by international regulatory authorities—demonstrated by numerous approvals of PLGA-based drugs [19–21]. PLGA is degrading delayed due to the hydrolysis of its ester linkages in presence of water. The resulting carboxylic acid chain ends of the cleavage further may further accelerate the process and lead to autocatalysis [22]. As a result of internal diffusion, a pH gradient from the center to the surface of the particle is forming and degradation products and will diffuse and erode away from the entire volume of the particle (bulk erosion). As PLGA degradation is driven by hydrolysis and degradation products (glycolic and lactic acid) are non-toxic chemicals that can be metabolized by the body, it can be used as delivery system in physiological environments [23]. Furthermore, PLGA is commonly used in the knee cavity for autologous chondrocyte transplantation, suture material and not known to interact with the joint tissue in any harmful way. Since the expiration of first patents on PLGA microparticulate systems, the development of generic products experiencing a revived interest in pharmaceutical research [24].

Advances in chemokine therapy are hampered by insufficient clinical biosafety data. Analysis of existing experimental data on CCL25 revealed strong involvement in adaptive immune system development, dysfunctional immunological processes in endometriosis, inflammatory bowel disease, and rheumatoid arthritis [25]. Hence, application of this chemokine could cause a significant inflammatory response and potentially cause more harm than good. Also, while the biocompatibility of PLGA is well established [26], there is currently no approved treatment that uses free-floating PLGA particles in human joints. It is also conceivable that the biomaterial itself could cause undesired inflammatory side effects related to its acidic properties.

To address these issues, we conducted an immunological study assessing the interaction of PLGA/CCL25 with different human immune cell subsets. The objective of this study was to assess the inflammatory potential of PLGA/CCL25 to induce typical changes that leukocytes show during an inflammatory response. In our analysis, we focused on the potential alteration of pro-inflammatory cytokine secretion, the regulation of cellular activation and polarization markers, and leukocyte migration induced by CCL25 or PLGA.

## Results

### Immune cell subsets exhibit differing expression levels of CCR9 

**Fig. 1:**
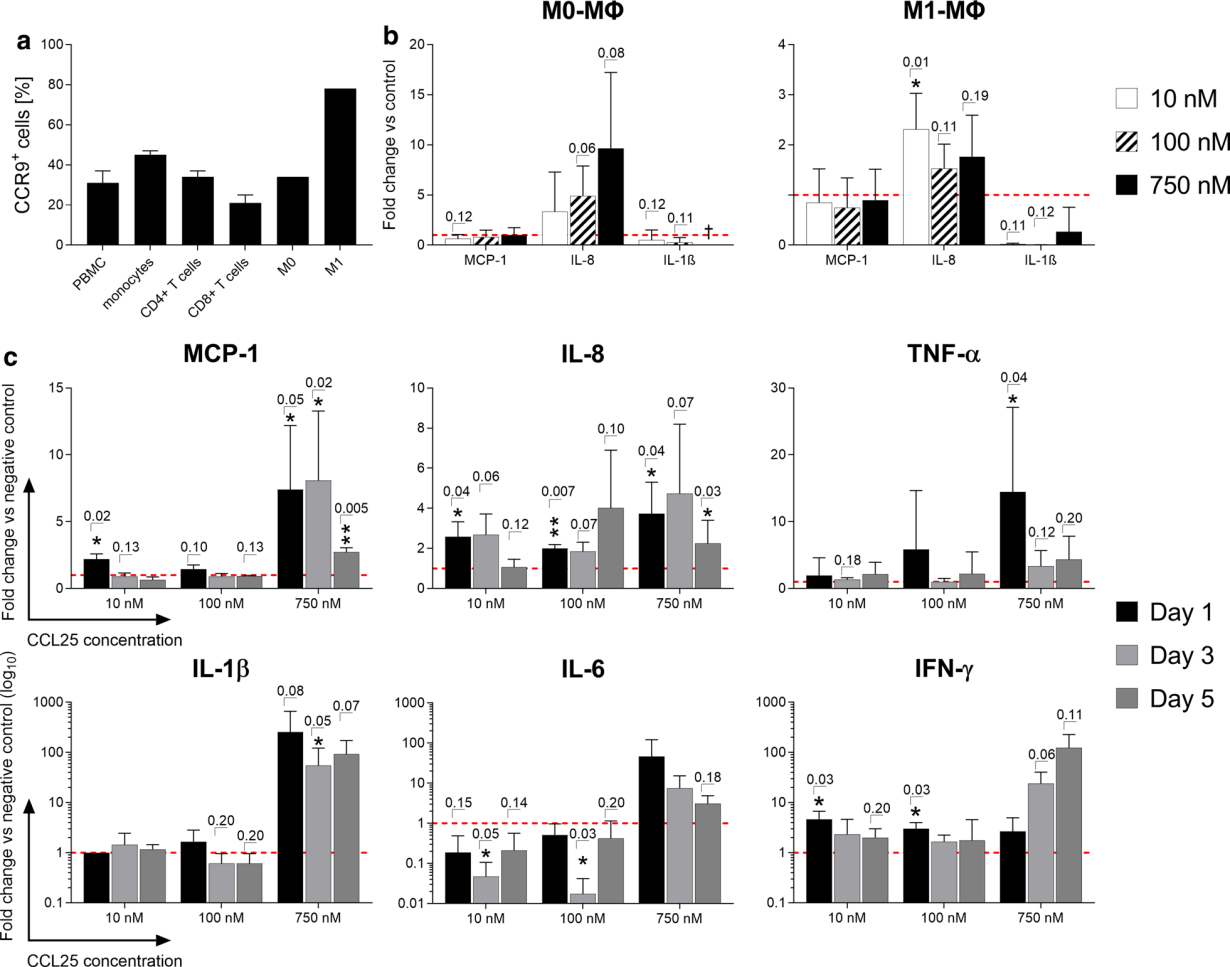
CCR9 expression and secretion profile of immune cell subsets after stimulation with CCL25. PBMCs, unpolarized (M0) and polarized macrophages (M1) were stained with a CCR9-specific antibody and analyzed by flow cytometry. PBMCs and macrophages were stimulated with different dosages of CCL25 or left unstimulated as a negative control. A LEGENDplex cytokine array was performed using the culture supernatants after day 1, day 3 and day 5. **a** The expression of the CCL25-receptor CCR9 on different immune cell subsets is shown as mean of the percentage of marker positive cells + SD (*n* = 1–4). **b** Shown are the cytokine/chemokine release data for M0 and M1 macrophages after one-day stimulation with 10, 100 and 750 nM CCL25 as the mean fold change + SD compared to the negative control (red dotted line) (*n* = 4). **c** The cytokine/chemokine release data for PBMCs stimulated with 10, 100 and 750 nM CCL25 over three days are displayed as the mean fold change + SD normalized to the negative control (red dotted line) (*n* = 3). Significance was analyzed using either a student’s T-test or the Mann–Whitney-*U* test depending on whether data followed normal distribution. Individual p-values are given above the bars if lower than *p* = 0.20. *PBMCs*:peripheral blood mononuclear cells, *ΜΦ* Macrophages

The determination of CCR9 expression is an important prerequisite for enabling CCL25-induced cell migration and intracellular signaling as it is the receptor for the ligand CCL25. Thus, extracellular CCR9 expression was investigated using flow cytometry on different immune cell subsets by staining with a fluorescently labeled antibody (Fig. 1a). Peripheral blood mononuclear cells (PBMCs), monocytes and CD4^+^ T-cells exhibited a mean CCR9 expression of 31 ± 6%, 45 ± 2% and 34 ± 3%, respectively. CD8^+^ T-cells showed the lowest mean surface expression, with 21 ± 4%. The strongest CCR9-expression was found on M1-type macrophages with around 78%. Unpolarized M0-type macrophages showed a seemingly lower CCR9 expression of about 34% (Fig. 1a).

### Stimulation with high dosages of CCL25 induces high secretion levels of pro-inflammatory cytokines in PBMCs

To assess the unmediated reaction of different leukocyte subsets to CCL25, PBMCs and differently polarized macrophages were stimulated over five days with dosages of 10, 100 and 750 nM CCL25 (Fig. 1b, c). Supernatants were collected on day one, three, and five, and the cytokines present were analyzed with the LEGENDplex immunoassay. When testing different macrophage subsets, unpolarized M0 macrophages reacted strongly and dose-dependently to CCL25 stimulation with an up to tenfold increase in IL-8 secretion in the 750 nM group *(p* = *0.08),* while M1-polarized macrophages showed a small increase independent of the dose used (Fig. 1b). Direct stimulation of PBMCs with CCL25 leads to a very strong and dose-dependent secretion of the pro-inflammatory chemokines and cytokines IL-8, MCP-1, TNF-α, IL-1β, interleukin-6 (IL-6) and IFN-γ (Fig. 1c). While for most cytokines/chemokines there were only moderate increases in the 10 nM and 100 nM CCL25 stimulation groups, PBMCs reacted with a strong increase in all tested mediators for the 750 nM stimulation group. While IL-8 also showed a statistically significant increase in secretion after being triggered with lower CCL25 concentrations, the other cytokines increased exclusively after stimulation with the highest CCL25 concentration. On day 5 of stimulation, secretion of cytokines and chemokines seemed to decline. Apart from IFN-γ, the cytokines measured on the fifth day showed lower values than on day 1 after stimulation.

### CCL25 causes a dose-dependent upregulation of the activation marker human leukocyte antigen-DR (HLA-DR) on immune cells

**Fig. 2:**
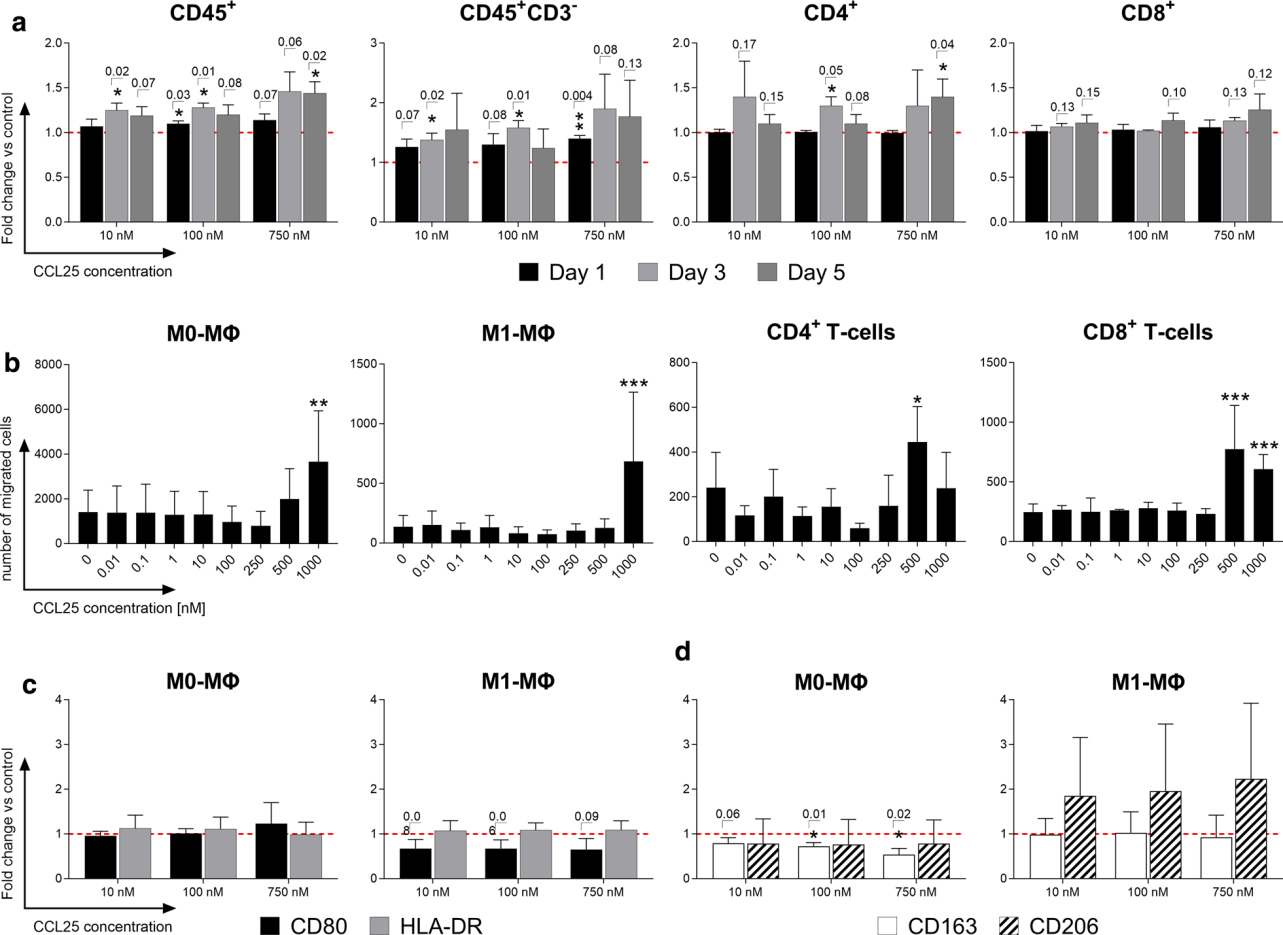
Surface marker changes and migration behavior of immune cell subsets after CCL25 stimulation. After stimulation with different dosages of CCL25 (10, 100, 750 nM), immunofluorescence staining with antibody panels and subsequent flow cytometry was performed to detect leukocyte activation and macrophage polarization based on surface marker expression. Additionally, a Boyden-Chamber migration assay was performed with a CCL25 dilution series (0.01–1000 nM). **a** HLA-DR expression levels of different immune cell subsets stimulated with 10, 100 and 750 nM CCL25 over three days are shown as the mean of the percentage of marker positive cells + SD normalized to the negative control (red dotted line); *n* = 3. **b** The migration of immune cell subsets towards a dilution series of CCL25 is displayed as the mean + SD of the number of migrated cells (*n* = 4). Macrophage polarization through CCL25 stimulation was analyzed by immunofluorescence antibody staining and flow cytometric analysis of M0 and M1 macrophages against either **c** M1 polarization markers CD80 and HLA-DR or **d** M2 polarization markers CD163 and CD206 (*n* = 4). Surface marker expression levels are shown as the mean + SD of normalized values compared to the negative control (red dotted line). Statistical significance was analyzed using either a student’s T-test or the Mann–Whitney-U test depending on whether data followed a normal distribution. Individual *p* values are given above the bars if lower than *p* = 0.20. *ΜΦ* Macrophages

Flow cytometry was used to investigate the effects of CCL25 on the activation and polarization of immune cells. Macrophages were first analyzed for the differential expression of characteristic polarization markers for the M1 (CD80 and HLA-DR) or M2 direction (CD163 and CD206). Analysis of the activation marker HLA-DR was then performed on PBMCs. While no statistically significant changes in the expression of either M1 or M2 type characteristic polarization markers on macrophages were detected (data not shown), PBMCs showed a very clear HLA-DR upregulation when exposed to all three concentrations of CCL25 (Fig. 2a). CD45^+^CD3^−^ negative cells (all mononuclear leukocytes besides T-cells) exhibited the strongest reaction, with a dose-dependent increase up to a 1.8-fold change compared to the negative control. Also, CD4^+^ cells reacted in a dose-dependent fashion with a significant HLA-DR upregulation to CCL25 stimulation. Cytotoxic CD8^+^ T-cells showed weaker changes in the expression level of HLA-DR than CD4^+^ cells and did not reach a statistically significant difference compared to the unstimulated negative control.

### Macrophages and T-cells show dose dependent migration towards CCL25

Assessing CCL25′s capabilities to recruit leukocytes from foreign tissues, different macrophage and T-cell subsets were examined in a Boyden-Chamber migration assay employing a CCL25 concentration range from 0.01 to 1000 nM (Fig. 2b). After CCL25 stimulation, both unpolarized M0- and M1-polarized macrophages reacted with a dose-dependent increase in migration. While migration towards CCL25 in unpolarized M0-macrophages already started at concentrations of 500 nM, M1 macrophages only showed significant migration towards the highest tested concentration of 1000 nM. T-cells also showed a strong increase in migration towards CCL25 concentrations of 500–1000 nM. The migration of CD8^+^ T-cells appeared to be stronger than that of CD4^+^ T-cells.

### Macrophages do not polarize or change their polarization upon CCL25 stimulation

M0- and M1-polarized macrophages were stimulated with 10, 100 and 750 nM CCL25, and then analyzed for their expression of surface markers specific for M1 or M2 macrophage polarization via flow cytometry (Fig. 2c). M0 macrophages showed no change in CD80 and HLA-DR (M1 markers), no significant change in CD206 and a significant decrease in CD163 (M2 marker) expression. In summary, no clear trend in the polarization mediated by CCL25 could be identified.

### Immune cell stimulation by CCL25-loaded and unloaded particles leads to increased IL-8 and MCP-1 secretion

**Fig. 3:**
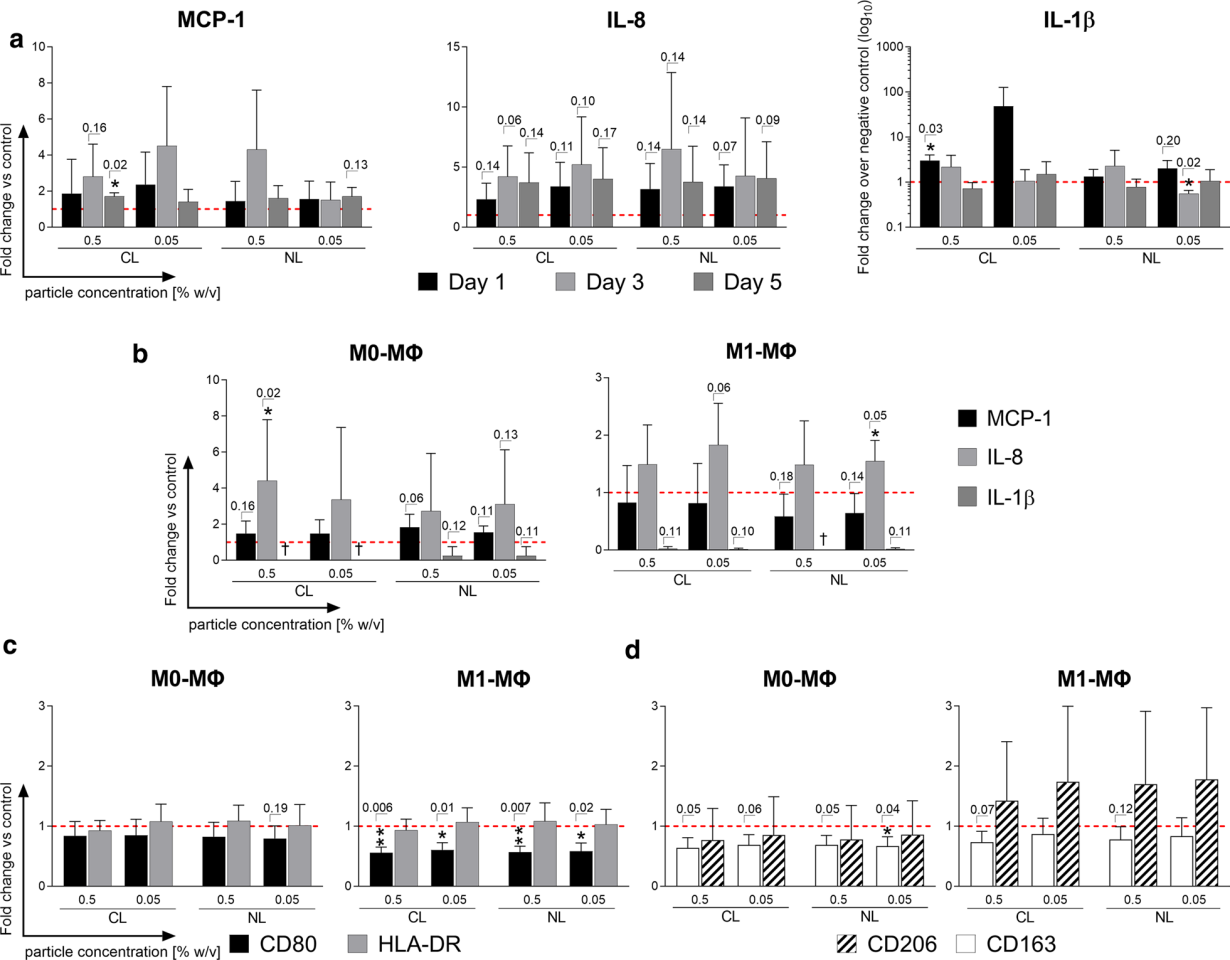
Response profile of immune cell subsets after stimulation with CCL25-loaded/unloaded PLGA particles. PBMCs and macrophages were stimulated with CCL25-loaded (CL) and unloaded (NL) PLGA particles at two different concentrations (0.5% w/v and 0.05% w/v). Induced changes in the cytokine/chemokine secretion profiles of PBMCs and macrophages were analyzed with a LEGENDplex cytokine array. In addition, unpolarized M0 macrophages and polarized M1 macrophages were stained for characteristic surface markers with fluorescently labeled antibodies and measured by flow cytometry to analyze their polarization status. **a** Cytokine release data (IL-8, *MCP-1, IL-1)* of PBMCs stimulated with 0.5% w/v and 0.05% w/v of CL and NL PLGA particles over 5 days are shown as mean fold change + SD normalized to the negative control (red dotted line) (*n* = 3). **b** Cytokine release data (IL-8, *MCP-1, IL-1)* of M0/M1 macrophages stimulated with 0.5% w/v and 0.05% w/v of CL and NL PLGA particles over 1 day are shown as the mean fold change + SD normalized to the negative control (red dotted line) (*n* = 4). Surface expression levels *of *M0 (left) and M1 (right) macrophages for the characteristic M1 polarization markers CD80 and HLA-DR (**c**) and for M2 polarization markers CD206 and CD163 (**d**) after stimulation with 0.5% w/v and 0.05% w/v of CL and NL PLGA particles over 1 day are displayed as mean of the fold change + SD normalized to the control (red dotted line), *(n* = *4).* Statistical analysis was performed using either a student’s T-test or the Mann–Whitney-U test depending on whether data showed normal distribution. Individual p-values are given above the bars if they are lower than *p* = 0.20*. CL* CCL25-loaded, *NL* non-loaded, *PC* Particle concentration., *w/v* weight per volume, *ΜΦ* Macrophages

Similar to the previous CCL25 stimulation assay, human leukocytes were incubated with undegraded CCL25-loaded (CL) and unloaded (NL) PLGA particles in two different concentrations (0.5% w/v and 0.05% w/v) to distinguish between the effect of CCL25 and intact particle, as well as to check the integrity of the loaded particles. Cytokine levels of PBMC cultures treated with both particle types increased for IL-8 and MCP-1 compared to the untreated negative control, peaking around the third day of stimulation (Fig. 3a). The strongest reaction for IL-8, albeit not statistically significant, was shown by the 0.5% w/v NL particles on the third day with a mean increase of 6.48 ± 6.38-fold change in secretion (*p* = *0.14).* In relation to the negative control, the mean increase in IL-8 for 0.5% w/v CL particles was, at 4.2, much lower than for the NL particles. Stimulation with 0.05% w/v of either CL or NL particles induced very similar fold changes for IL-8 secretion with the CL particles causing a slightly higher IL-8 secretion on the third day than the NL particles (CL = 5.2 ± 3.98; NL = 4.26 ± 4.84). Interestingly, all stimulation groups reached a very similar level of IL-8 secretion on the fifth day, with around four times that of the negative control. For MCP-1, all groups except the 0.05% w/v concentration of the NL particles showed high increases in secretion on the third day. The highest mean increase was seen in 0.05% w/v CL particles (4.5 ± 3.3-fold change; *p* = *0.22)* followed by the 0.5% w/v NL particles (4.3 ± 3.3-fold change; *p* = *0.35*). As seen with IL-8, MCP-1 secretion on day 5 equalized to similar levels at about 1.5-fold of the negative control, but only the 0.5% w/v concentration of the CL particle reached statistical significance (1.7 ± 0.5-fold change*; p* = *0.02).* Macrophages also responded to CCL25 stimulation by increasing secretion of IL-8 but did not increase secretion of MCP-1 or other cytokines (Fig. 3b). M1-polarized macrophages reacted similarly to 0.5% and 0.05% w/v of either CL or NL particles, with a slight increase in IL-8 secretion (CL*: 0.5% w/v*: 1.49 ± 0.68; *0.05% w/v*: 1.48 ± 0.76; NL: *0.5% w/v*: 1.83 ± 0.72; *0.05% w/v*: 1.55 ± 0.35). Unpolarized M0 macrophages reacted with a much stronger overall secretion of IL-8 and an observable difference between CCL25-loaded and unloaded particles (CL: *0.5% w/v:* 4.42 ± 3.4; *0.05% w/v*: 3.37 ± 4.0; NL: *0.5% w/v*: 2.73 ± 3.2; *0.05% w/v*: 3.11 ± 3.0).

### Both CCL25-loaded and unloaded particles modulate the M1 macrophage phenotype

Next, we determined whether the unloaded or CCL25-loaded particles could influence the polarization status of human macrophages in vitro and even reverse the development of a pro-inflammatory M1 phenotype. Both the CL and NL particle groups caused a significant reduction of CD80 expression, a typical M1 marker, in cultures of M1-polarized macrophages by a mean of 1.52 ± 0.07 (Fig. 3c). However, the second M1 marker assayed, HLA-DR, showed no significant changes in expression level. At the same time, expression of the characteristic M2 surface marker CD206 in co-cultures of already polarized M1 macrophages with the particles trended towards an increase with a fold change of 1.50 ± 1.57. Expression of the M2 Marker CD163 trended towards a decrease of around 1.20 in both groups with the 0.5% w/v particles reaching p-values of 0.07 and 0.12, respectively (Fig. 3d). Unpolarized M0 macrophages also showed a slight reduction of CD80 and CD163 after particle incubation, but no increase in CD206 (Fig. 3b, d). No significant differences between the loaded and unloaded particles were observed.

### Immune cell stimulation with supernatants of CCL25-loaded particles causes an increase in IL-8, MCP-1 and IL-1β secretion

**Fig. 4:**
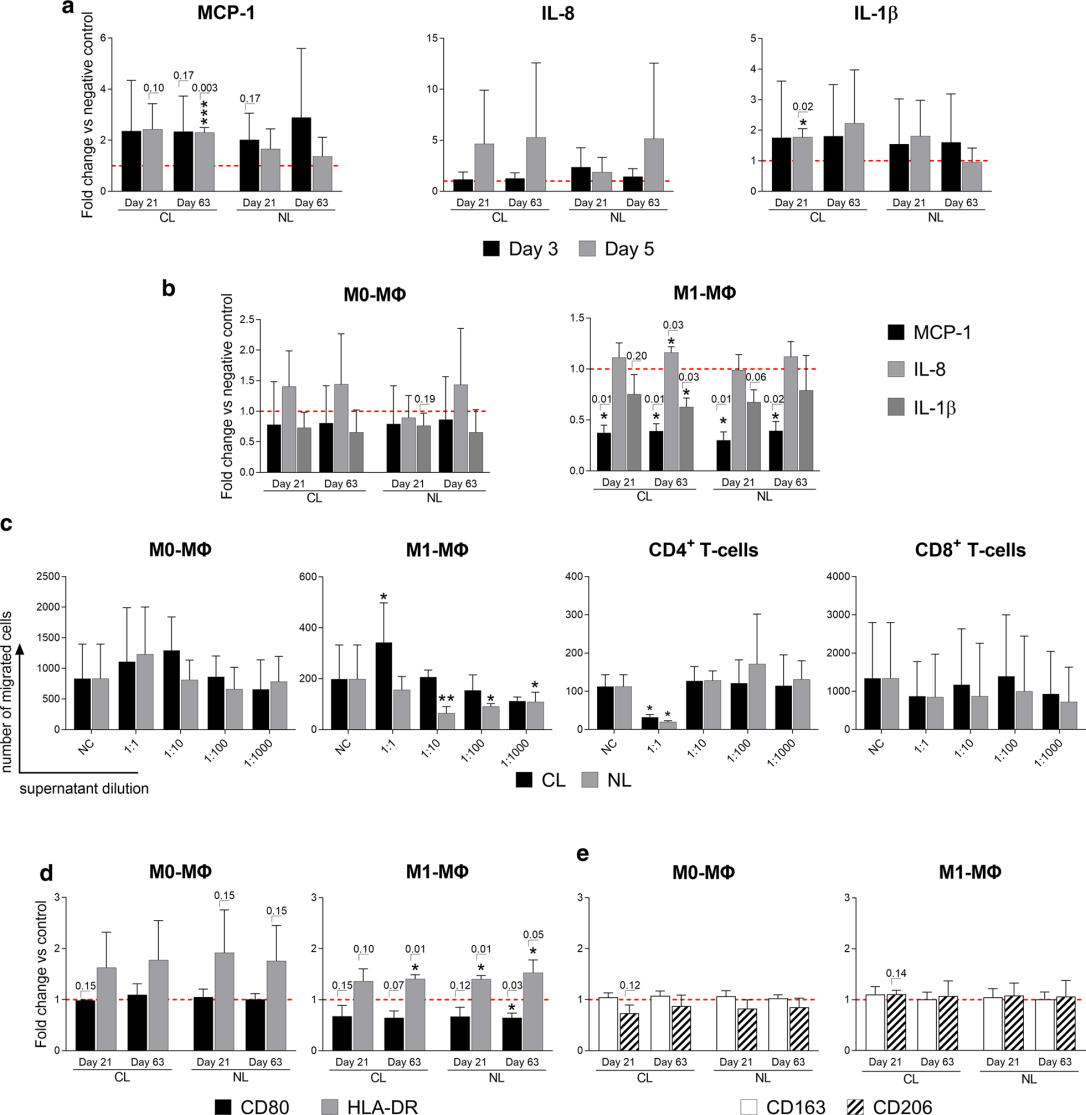
Reaction of immune cell subsets after stimulation with supernatant from degraded CCL25-loaded/unloaded PLGA particles. CCL25-loaded (CL) and unloaded (NL) PLGA particles were allowed to degrade for either 21 or 63 days and particle supernatant was collected and used for in vitro stimulation assays with human PBMCs and macrophages. Changes in the cytokine/chemokine release pattern of PBMCs and macrophages were analyzed with a LEGENDplex cytokine array. Additionally, macrophages were analyzed by immunofluorescence staining and flow cytometry for the expression of surface markers indicating activation or polarization. **a** Cytokine array of PBMCs stimulated over 5 days with 21- and 63-day CL and NL PLGA particle supernatants. Cytokines with detectable differences in fold change are IL-8, *MCP-1 and IL-1β.* Data is shown as the mean fold change + SD normalized to the negative control (red dotted line) (*n* = 3). **b** Cytokine array of M0/M1 macrophages stimulated over 1 day with 21- and 63-day CL and NL PLGA particle supernatants. Cytokines with detectable differences in fold change are IL-8 and MCP-1*.* Data is shown as mean fold change + SD normalized to the negative control (red dotted line) (*n* = 4). **c** The migration of immune cell subsets towards a dilution series of particle degradation supernatant is displayed as the mean + SD of the number of migrated cells (*n* = 4). **d/e*** Flow cytometric analysis of M0/M1 macrophage polarization surface markers after* 1 day *of stimulation* with 21- and 63-day CL and NL PLGA particle supernatants. Increased CD80 and HLA-DR indicates M1 polarization (d), while increased CD163 and CD206 indicates M2 polarization (e). Results are displayed as mean of the fold change + SD normalized to the control (red dotted line), *(n* = *4).* Statistical analysis was performed using either a student’s T-test or the Mann–Whitney-*U* test depending on whether data followed normal distribution. Individual *p*-values are given above the bars if lower than *p* = 0.20. *CL* CCL25-loaded, *NL* non-loaded, *NC* negative control, *ΜΦ* Macrophage

To distinguish between the effects of particle fragments and the enclosed CCL25 on the leukocytes, CL and NL particles were both allowed to degrade over either 21 or 63 days. The supernatants of degraded particles were applied to the PBMCs in functional in vitro assays, and the cytokine secretion profile was determined at day 3 and 5 after supernatant stimulation (Fig. 4a). PBMC cultures treated with particle supernatants showed higher secretion levels of IL-8, MCP-1 and IL-1β compared to the control. In the CL group, IL-8 secretion was increased 4.6-fold (± 5.3, *p* = 0.22) after treatment with the 21-day supernatant and 5.3-fold (± 7.3, *p* = 0.45) with the 63-day supernatant. The latter NL supernatants only induced a 5.14-fold (± 7.4, *p* = 0.49) increase in IL-8 levels, which does not reach statistical significance. IL-1β secretion increased in a statistically insignificant manner by roughly the same amount in the CL and NL particle groups after three days (CL: *21d*: 1.75 ± 1.85; *63d*: 1.81 ± 1.68; NL: *21d*: 1.54 ± 1.47; *63d*: 1.6 ± 1.58). However, after five days, secretion of IL-1β in PBMCs stimulated with the 21-day CL supernatant increased by a statistically significant amount (1.77 ± 0.27: *p* = *0.02*), while for the corresponding NL particle supernatants, the IL-1β secretion tended to drop down to the levels of the negative control (0.95 ± 0.46*)*. The same dynamics were observed for MCP-1 secretion, with a strong increase after three days in all groups, and with high levels maintained on the fifth day in the CL particle supernatant group. At the same time, MCP-1 secretion decreased in the NL particle supernatant group (Fig. 4a). Again, macrophages were also analyzed in the same way, but only showed higher secretion levels for IL-8. The 21-day and 63-day old CL particle supernatants caused a 1.4-fold (± 0.5, *p* = *0.32*)/1.43 (± 0.83, *p* = *0.44*) increase in cultures of M0 macrophages and a 1.15 (± 0.29, *p* = *0.43*) / 1.29 (± 0.04, *p* = *0.03)* increase in M1 macrophages, respectively (Fig. 4b). Interestingly, IL-1β secretion from M1-polarized macrophages was significantly lower in all CL and NL supernatant groups.

### Supernatants of both loaded and unloaded particles cause no significant increases in immune cell migration

To assess the chemoattractive potential of the particle components, the supernatants of degraded particles (21 and 63 days) were used as stimuli in a Boyden-Chamber migration assay with the different leukocyte subsets (Fig. 4c). Stimulation was performed in a 1000-factor dilution series to distinguish between the effects of CCL25 and PLGA fragments. When testing CD4^+^ T-cells, undiluted supernatants of both particle groups reduced the migration tenfold, which is a statistically significant decrease. Also, M1 macrophages showed a statistically significant reduction in migration at 1:10 and 1:100 dilutions of the particle supernatants, but only in the NL group. The other tested immune cell subsets (M0 macrophages, CD8^+^ T-cells) showed no significant differences in migration in response to the particle supernatants compared to the control (Fig. 4c).

### Supernatants of both loaded and unloaded particles increase HLA-DR expression on macrophages

To assess the capacity of the particle supernatants to modulate the activation or polarization state of human macrophages, the surface expression levels of the characteristic markers were analyzed by flow cytometry. After treatment of M1-polarized macrophages with CL and NL particle supernatants, HLA-DR expression was significantly increased with a mean fold change of 1.4 ± 0.2 (Fig. 4d). However, there was no observable difference between the groups treated with CCL25-loaded- and unloaded particle supernatant and the timespan of degradation. Meanwhile, the M1 marker CD80 decreased by a mean fold change of 1.3 (± 0.17). M0-polarized macrophages also increased expression of HLA-DR after supernatant stimulation from either CL or NL particles, but did not reach statistical significance. However, changes in the expression level of the M2 markers CD206 and CD163 induced by the particle supernatant treatment were not observable in either M0 or M1 phenotypes (Fig. 4d).

## Discussion and limitations

The objective of this study was to analyze the inflammatory potential of a novel therapy for OA based on CCL25-loaded PLGA particles. We wanted to examine the reaction of leukocytes involved in innate and adaptive immune responses towards each individual component of the potential therapeutic agent to better assess which subcomponents are potentially critical and at what doses. For this purpose, human PBMCs isolated from whole blood and macrophages generated in vitro were exposed to different dosages of (i) pure CCL25, (ii) CCL25-loaded PLGA particles and (iii) their supernatants after varying time points of particle degradation. We subsequently analyzed the changes in pro-inflammatory cytokine secretion, the leukocyte activation and macrophage polarization by monitoring characteristic cell surface markers and investigated immune cell migration. Our results provided new insights regarding the inflammatory potential of a possible PLGA-based delivery chemokine therapy for the treatment of OA. Crucially, the CCL25 receptor CCR9 was found to be expressed on all analyzed leukocyte subsets, which makes a reaction upon contact with this chemokine very likely and underlines the need for a thorough analysis of this interaction.

The dose-dependent reaction of different leukocyte subsets to stimulation with CCL25 was remarkably clear. While the immune cells reacted only slightly to CCL25 concentrations of 10 and 100 nM, stimulation with 750 nM CCL25 caused a strong secretion of MCP1, IL-8, TNF-α, IL-1β, IL-6 and IFN-γ from PBMCs. This combination of cytokines indicates a general, uncontrolled leukocytic activation. Except for IFN-γ, secretion levels of all cytokines start to drop down after 5 days of stimulation. However, these cytokines were considered to be among the main biological drivers in the pathogenesis of OA. Especially MCP-1 and IL-8 are reported to correlate with clinical levels of pain and knee swelling. Concerningly, these two cytokines exhibited the largest increase in expression for all dosages of CCL25 in both PBMCs and macrophages [27–29]. In an in vivo situation, this high level might subsequently cause an increase in inflammation by damaging the surrounding tissue and possibly further recruiting more immune cells to the site. Flow cytometric analysis of HLA-DR expression revealed a high sensitivity, especially for monocytes and CD4^+^ T-cells, to CCL25 stimulation, with visible increases in expression even at low concentrations of 10 nM CCL25. This most likely contributes to the strong cytokine secretion in PBMCs through the simultaneous activation of both monocytes and CD4^+^ T-cells, resulting in an amplification effect. This would also explain why macrophages alone do not respond as strongly as PBMCs, as they were cultured in isolation from other cell types and probably not exposed to a strong paracrine co-stimulus. CCL25 also exhibited the critical potential of recruiting leukocytes, which was expected given the physiologic role of CCL25 in T-cell homing [30]. Previous studies already demonstrated the capability of injected CCL25 to recruit T-cells to the site of injection [31]. Beginning at a certain concentration, unpolarized M0 macrophages and M1 polarized macrophages as well as CD4^+^ and CD8^+^ T-cells started to strongly migrate towards CCL25 concentration gradients. The critical dose for migration in vitro was between 500–1000 nM, which roughly corresponds to the concentrations that also triggered cytokine secretion. The level of HLA-DR expression induced by CCL25 stimulation correlates with the CCR9 expression profile, with monocytes revealing the highest CCR9 expression and HLA-DR upregulation, while CD8^+^ T-cells showed the lowest CCR9 expression and HLA-DR upregulation. It is also likely that the strong HLA-DR upregulation observed is due to co-stimulation by secreted cytokines. Since the macrophages were cultivated as monocultures, they could not acquire a co-stimulus from other leukocytes. This might explain that they show no HLA-DR upregulation, despite carrying high amounts of CCR9 on their surface. In summary, CCL25, starting at concentrations of 250–500 nM, has a strong potential to trigger inflammatory processes by recruiting and activating leukocytes and inducing cytokine secretion, especially in a heterogeneous cell environment such as chronically inflamed tissue or blood.

When analyzing the immune cell responses to the CCL25-loaded PLGA particles, the induced response was much weaker compared to the direct CCL25 stimulation. The CCL25-loaded particles themselves triggered the release of cytokines such as IL-8, MCP-1 and IL-1β in PBMCs, and IL-8 in the M0 and M1 macrophage subsets. The increase of IL-8 and MCP-1 was slightly stronger and achieved more statistical significance in the CCL25-loaded particle group. IL-1β only increased on the first day of stimulation with loaded particles. Overall, however, the difference between the loaded and unloaded particles was minute. It is very likely, that the reaction was mostly caused by the PLGA, with a marginal CCL25 contribution. The observed increase in IL-8 secretion could be induced by the acidic degradation of the PLGA, which has been shown to cause IL-8 production in other cells [32, 33]. The influence of CCL25-loaded particles on the polarization of macrophages did not show a very clear polarization trend towards either a more pro-inflammatory M1-type or the pro-regenerative M2-type. However, the decrease of CD80 and increase of CD206 point in the direction of increased M2 polarization. Macrophage polarization towards the pro-regenerative M2 macrophage direction by PLGA has been recently described by others [34, 35]. M2 macrophage polarization is thought to be a highly favorable feature of tissue engineering devices to decrease inflammation and promote healing [36, 37]. This in turn underlines the suitability of PLGA as a delivery platform for regenerative therapeutics. Overall, these results are in line with the existing literature. PLGA has been used clinically as a surgical suture material for years and is not known to cause major inflammatory side effects other than the aforementioned acidic degradation. On the contrary, the promotion of M2 macrophage polarization might even prove advantageous to minimize an already existing inflammation.

**Fig. 5:**
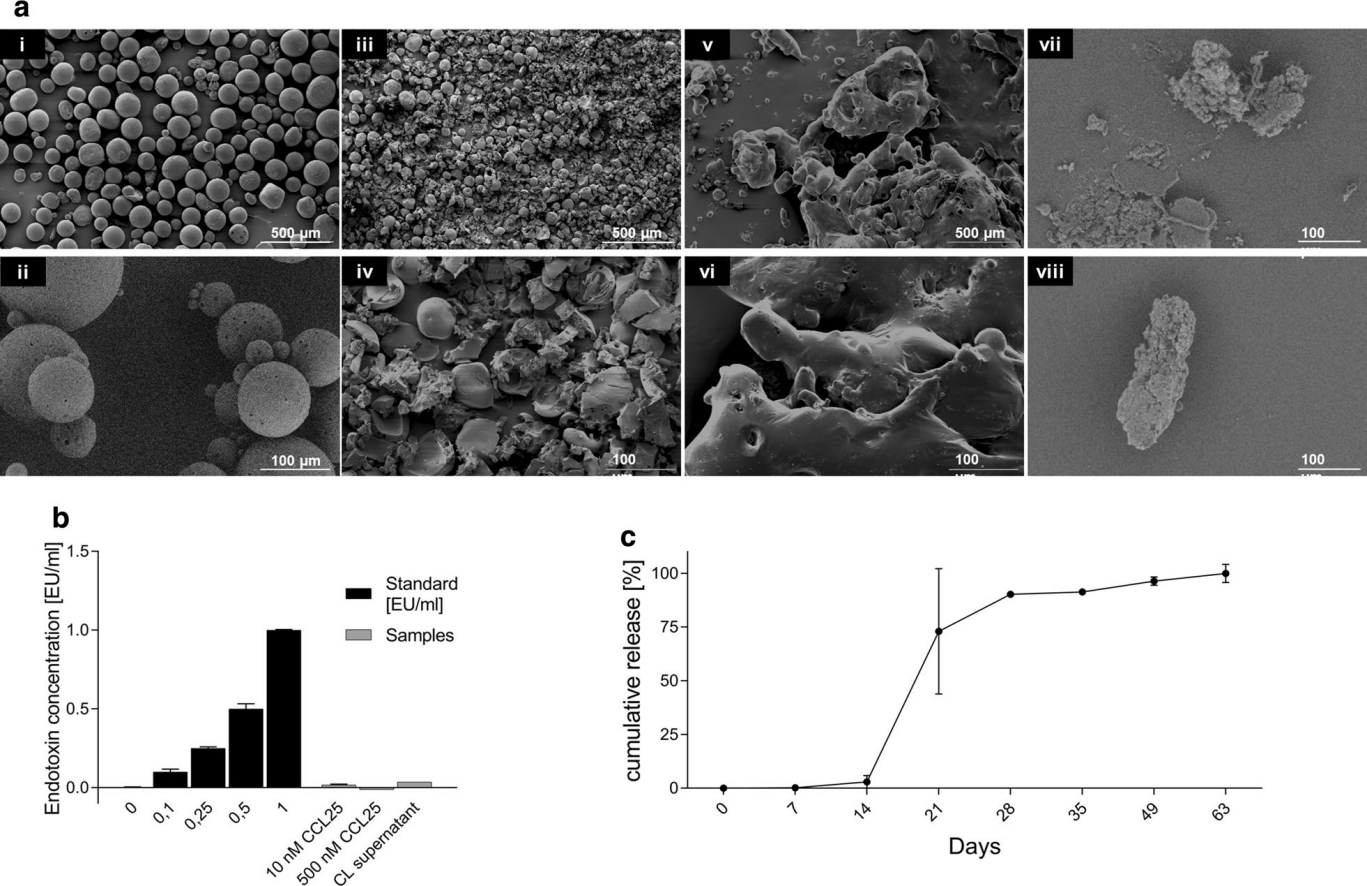
Characteristic features of PLGA particles. PLGA particles were analyzed morphologically with SEM, evaluated for endotoxin contamination with the Limulus amebocyte lysate (LAL) test, and functionally assessed by their CCL25 release profile. Representative SEM images of the PLGA particles generated are shown in **a** **i,ii** with × 40 and 200 magnification at day one, **iii, iv** with × 40/ × 200 magnification after 14 days of degradation, **v, vi** with × 40/ × 200 magnification after 28 days of degradation and **vii, viii** with × 1000 magnification after 63 days of degradation. Scale bars represent 500 and 100 µm, respectively. **b** The results of the LAL test for the detection of endotoxins are shown for different stock solutions of CCL25 (10 and 500 nM) and the supernatant from PLGA particles (CL supernatant) compared to the test standards (0.1–10 nM). **c** The cumulative release in percent ± SD of CCL25 from PLGA particles over time until day 63 (*n* = 3) was determined by ELISA. *CL* CCL25-loaded, *PLGA* Poly (lactic-co-glycolic acid)

After stimulation with supernatants from the degraded CCL25-loaded particles, PBMCs showed a much stronger reaction induction of cytokine secretion than after the stimulation with undegraded but loaded particles. Considering the release pattern exhibited by the particles (Fig. 5c), supernatants at day 21 and 63 contain roughly 75% and 100% of released chemokine, respectively. This amounts to a maximum of 352 nM (63 days) 35.2 nM (21 days) of CCL25. The supernatants contained both the degraded PLGA fragments and the chemokine CCL25, and triggered release of IL-8 and MCP-1 by immune cells as well as IL-1β in addition to the particles. Notably, the supernatants of CCL25 loaded particles caused a higher and longer secretion of these cytokines. IL-1β is a highly potent pro-inflammatory cytokine, of which only a few nanograms are sufficient to cause massive local inflammation and tissue degradation, especially in human joint tissue [38–41]. These findings must be considered in the context of the in vivo situation. CCL25 as well as PLGA would also be degraded enzymatically over time, meaning that particle supernatants generated in vitro contained much more CCL25 and undegraded fragments than would occur in a therapeutic situation. Further, compared to the direct stimulation with CCL25, the supernatants of the degraded particles did not cause an increase in HLA-DR expression on immune cells or enhance their migration. This does suggest that, even though the theoretical concentration of CCL25 in the supernatants amounted up to 352 nM, due to the protein degradation (average half-time of proteins in aqueous phases is about 5–7 days) [42] the proportion of active CCL25 always remains below the presumed critical concentration of 100 nM where definitive inflammatory activation of immune cells becomes visible. However, the concentration of CCL25 previously applied in vivo and identified as beneficial was approximately 1–5 nM [13]. Concerning cytokine release and leukocyte chemoattraction, the first concentrations which prove to be critical lie slightly above 100 nM. Our findings suggest that the intra-articular concentration should never exceed these values, which can be assured by controlled release from an implant with a slow release kinetic. Since the leukocyte responses to CCL25-loaded PLGA particles that were observed in vitro did not differ much from the unloaded particles, it can be expected that particles carefully loaded with CCL25 are most likely safe to use. In fact, the secretion of IL-8 and MCP-1 after stimulation of leukocytes by polylactic scaffolds has been previously described by Caires et al. [43] as potentially beneficial for healing by aiding in MSC recruitment. Nevertheless, it must be assured that the release device used is tear resistant. A sudden release of large amounts of CCL25 could prove highly counterproductive in the therapy of OA, as it increases rather than reduces intraarticular inflammation.

The strength of this study is the broad spectrum of different analytic methods and leukocyte subsets employed, which made it possible to draw a differentiated picture of the inflammatory potential of the different components of a CCL25-loaded particle for potential OA treatment. The study is limited by the relatively low number of human immune cell donors and the strong variability which impeded the generation of statistically valid statements due to high standard deviations. Nevertheless, the analysis of different leukocyte subgroups as well as differently polarized macrophages allowed detailed insights into the type and extent of the expected inflammatory reaction and sheds light on the complex mechanisms of signal transduction of the chemokine CCL25 in the context of a potential clinical application.

## Conclusion

Delayed intra-articular release of CCL25 is a promising approach for a regenerative therapeutic to treat cartilage degeneration in OA. PLGA appears to have only mild few pro-inflammatory properties and may even induce beneficial M2 macrophage polarization. However, high doses of CCL25 indeed do carry the risk of inducing an extensive inflammatory response by immune cells present in joint tissue. We demonstrated for the first time, that externally applied CCL25 is capable to induce secretion of proinflammatory cytokines, including TNF-α, IL-1β, and IFN-γ in PBMCs, HLA-DR upregulation on monocytes and CD4 + T cells, and enhanced migration of CD4 + T-cells and macrophages. Although the previously determined targeted therapeutic doses of CCL25 of about 1 nM are well below the measured inflammatory cutoff of 100 nM—spontaneous disruption of the particles could release a large amount into the joint cavity and produce serious side effects. Due to these potentially dangerous side effects, our results suggest very high-quality requirements for any CCL25 delivery device. Essentially, each batch must be tested for structural integrity in the frame of tests for spontaneous degradation and resistance to mechanical stress.

## Methods

### Production of CCL25-loaded PLGA microparticles and sampling

To enclose the hydrophilic chemokine CCL25 (Peprotech, Rockyhill USA) within the biodegradable polymer PLGA (Sigma-Aldrich, St. Louis USA), a water-in-oil-in-water (w_1_/o/w_2_) double emulsion with subsequent solvent evaporation was performed according to a previously published method [15]: PLGA polymer (Resomer®, RG 503—Evonik Industries, Essen, Germany) was dissolved (100 mg polymer in 0.7 ml methylene chloride) and served as organic (o) phase. The internal aqueous phase (w1) was comprised of 1 µg CCL25 per mg PLGA that was dissolved in 66.5 ml lactose (300 mM)/BSA (0% or 5% w/w)/Tris–HCl (10 mM)-EDTA (1 mM) buffer. For preparation of the w1/o primary emulsion, the w1-phase was emulsified in the o-phase either by vortex mixing at 2500 rpm for 1 min or by sonication for 10 s at 7 W. For fabrication of big-sized microparticle formulations, the primary emulsion was subsequently emulsified into 80 ml of the external aqueous w2 phase (5% (w/v) poly(vinylalcohol) (PVA,MW 67,000, Sigma-Aldrich)). In order to yield small-sized microparticles of formulation, the primary emulsion was first emulsified in 2 ml 5% PVA by vortex mixing at 2500 rpm for 3 min and the resulting w1/o/w2 double emulsion was further poured into 30 ml 1% PVA. The double emulsions were stirred continuously for 3 h for solvent evaporation. The solidified microparticles were centrifuged and rinsed three times with distilled water. In addition to CCL25-loaded particles (CL-particles), a batch with non-loaded particles (NL-particles) was also produced to serve as negative control. After production, the particles were freeze-dried over-night and stored at 4 °C. The release samples were generated by incubating the particles at 37 °C in a shaker at 25 rpm. Sampling was performed after centrifugation for 5 min at 600*g* by collecting the supernatant, which was stored at −20 °C. The samples were transferred into pre-weighed tubes and the collected volume was determined with a precision scale. It was assumed that 1 g supernatant equaled 1 ml. After collection of the supernatant, fresh medium was added to the particle solution, and they were placed back in the shaker. The samples were collected every seven days beginning with day 7 and every 14 days from day 35 to day 63.

### Morphological analysis of PLGA particles

To ensure sufficient quality of produced particles, size and surface morphology was analyzed using light microscopy and scanning electron microscopy (SEM). A small amount of each PLGA batch was dispersed in Aquatex™ (Millipore Sigma, Burlington USA) and 100 to 500 particles were photographed. Light microscopy images were captured with the software ProgRes Capture Pro™ (Jenoptik, Jena, Germany) and analyzed in ImageJ (Version 1.8.0.112). The diameters of 10%, 50%, 90% and 99% (D_V10_, D_V50_, D_V90_, D_V99_) of the particles were obtained. D_V50_ of produced particles was at ~ 84 µm, which is within the previously reported range of optimal size for maximal CCL25 release [15]. Pictures of SEM were taken with an SEM JFM-6000 electron microscope (Jeol, Akishima, Japan) at day one and day 63 using the backscattered electrons (BSE) detector at 200× and 1000× magnification. SEM analysis revealed round particles, which exhibit few pore formations on an otherwise smooth surface. After 63 days, the shape of the previously round particles was completely lost, leaving only fragments or deformed particles, showing the degradation process is a requirement for functioning CCL25 release (Fig. 5a).

### Endotoxin detection

Immune cell reactions are highly inducible by endotoxins. To determine if cellular reactions to all tested samples (CCL25, CCL25-loaded PLGA particles and supernatants of CCL25-loaded PLGA particles) would be distorted by the presence of endotoxins, an endotoxin assay was performed with all stimulation components (10 nM and 500 nM stock solution; supernatants of particle release) (Fig. 5b). Gram-negative bacterial endotoxin was analyzed by the Pierce™ LAL Chromogenic Endotoxin Quantitation kit (Thermo Fisher Scientific, Waltham, USA) according to manufacturer instructions. Absorbance at 410 nm was measured on a SpectraMax™ plate reader (Molecular Devices, San Jose, USA). All values remained under 0.1 EU/ml, showing the absence of endotoxin in the samples.

### CCL25 determination

The amount of CCL25 released from the PLGA microparticles was measured using the human CCL25/TECK DuoSet® Enzyme linked immunosorbent assay (ELISA) kit (R&D Systems, Minneapolis, USA). The optical density was measured at 450 nm and 570 nm using the Synergy™ HT Multi-Detection Microplate Reader (BioTek, Winooski, USA; Fig. 5c). The optical imperfections in the plate were corrected for by subtracting the background at 570 nm from the readings at 450 nm. The data were evaluated considering the qualitative (blank plus three times the standard deviation of the blank) and quantitative (blank plus nine times the standard deviation of the blank) borders of the ELISA. CCL25 release started slowly on day 14, with more than 90% of total CCL25 detected between day 21 and day 28. The strongest release was observed between days 14 and 21. Recovery of CCL25 amounted to 77% of incorporated CCL25.

### Isolation and cultivation of human immune cell subsets

Peripheral blood mononuclear cells (PBMCs) were isolated from buffy coats and human peripheral blood from healthy volunteers using protocols approved by the ethics committee of the Charité Universitätsmedizin Berlin (EA2/139/10; EA1/226/14) via density gradient centrifugation using Biocoll (BioTek, Winooski, USA). Lymphocytes and monocytes were isolated from PBMCs via positive selection with antibody-labeled magnetic MicroBeads (Miltenyi Biotec, Bergisch Gladbach, Germany) and MACS-columns. 20 μl CD4, CD8 (for the isolation of CD4^+^ and CD8^+^ T-lymphocytes, respectively) or CD14 (for the isolation of monocytes) MicroBeads per 10^7^ cells were added to label the corresponding cell subtypes. To generate unpolarized (M0) macrophages, CD14^+^ monocytes were resuspended in complete cell culture medium (VLE-RPMI, 0.1% AB serum, 1% Penicillin/Streptomycin) containing M-CSF (50 ng/ml Miltenyi Biotec, Bergisch Gladbach, Germany), seeded into 6-well cell culture plates, and incubated at 37 °C and 5% CO_2_. After six days of stimulation in culture, monocytes were differentiated into M0 macrophages. To polarize them further towards pro-inflammatory M1 type macrophages, the cells were detached from the cell culture plates using 0.5 ml Accutase (Innovative Cell Technologies Inc., San Diego, USA). Then, two million cells were seeded in 6-wells in complete medium supplemented with 20 ng/ml IFN-γ (Peprotech, Rocky Hill, USA) and 100 ng/ml lipopolysaccharide (LPS; Sigma-Aldrich, St. Louis–USA) to enable polarization within 48 to 72 h towards the M1 macrophage type.

### Stimulation of immune cell subsets

For the analysis of the immune cell secretome, PBMCs of three different donors were seeded in 6-well plates, containing two million cells per well in 2 ml of cell complete culture medium. Cells were incubated with the following stimuli: (i) different concentrations of CCL25 (10, 100 and 750 nM), (ii) 10 and 1 mg of CCL25-loaded and unloaded particles, and (iii) the supernatants of loaded and unloaded particles that were previously degraded over 21 or 63 days (5 mg/ml). Assuming full degradation, the maximum achievable concentration in (ii) would amount to 352 and 35.2 nM, respectively. Regarding (iii), the theoretical concentration of CCL25 in the supernatants would amount to 75% of total released chemokine at 21 days, and 100% at 63 days (Fig. 5c). This is also why these timepoints were chosen for the supernatants. One well was always left unstimulated to serve as a negative control. 200 µl of cell culture supernatants were harvested and stored after 1, 3 and 5 days for further analysis at − 80 °C. The same stimulation setting was used for the macrophages, which were only cultivated for 48 h, since expression profiles of macrophages already change after 24 h of culture. For the flow cytometric analysis of PBMCs, 0.2 million cells were seeded in 96-well plates containing 200 µl of complete cell culture medium.

### Analysis of surface marker expression on immune cells by flow cytometry

**Fig. 6:**
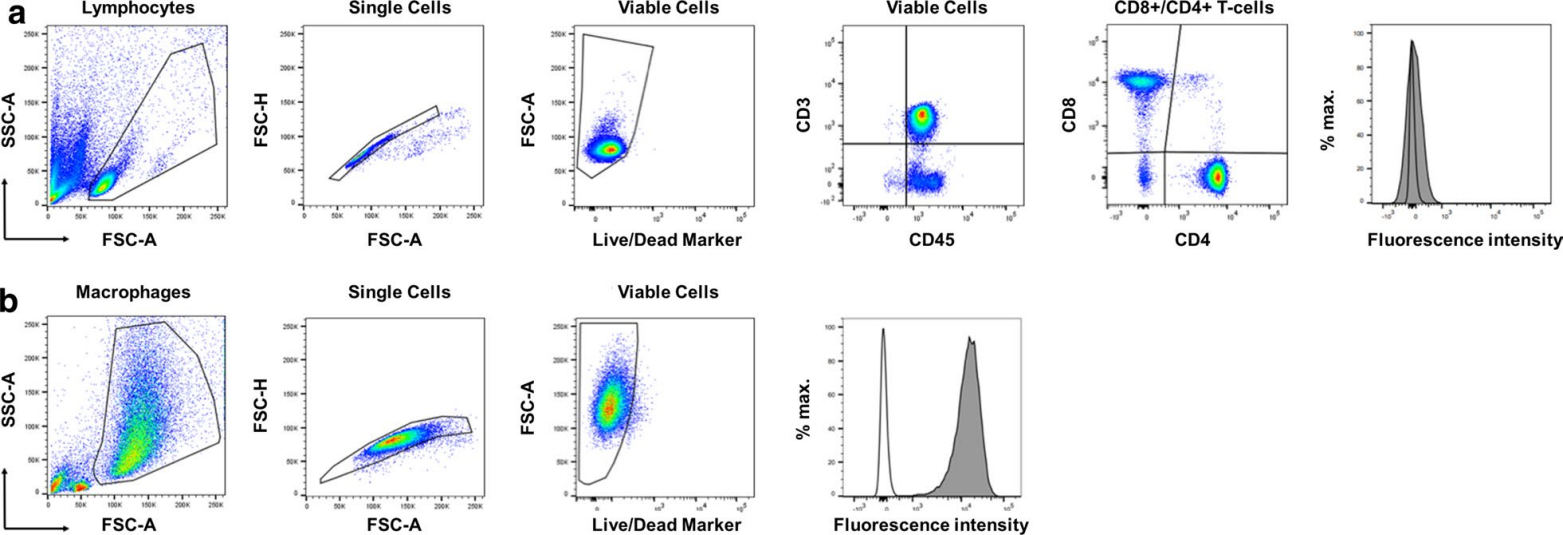
Gating strategies for the surface marker analysis of human peripheral blood mononuclear cells (PBMCs) (**a**) and macrophages (**b**). **a** PBMCs were first gated (black lined gate) on via sideward scatter area (SSC-A) and forward scatter area (FSC-A) on lymphocytes. Doublets were excluded by gating on forward scatter height (FSC-H) vs. FSC-A and then viable cells were gated by FSC-A vs. Live/Dead Marker. Thereafter, CD3 + CD45 + T cells were identified based on gating by CD3 vs. CD45, which were then separated into CD4 + and CD8 + T cells. Finally, the mean fluorescence intensity (MFI) of the marker of interest (filled grey histogram) was determined in comparison to the negative control staining (black lined histogram). **b** Macrophages were first gated via SSC-A vs. FSC-A to exclude cell debris from the analysis. Doublets were excluded by gating on FSC-H vs. FSC-A. Then, viable cells were identified (FSC-A vs. Live/Dead) and the MFI of the marker of interest (filled grey histogram) was determined in comparison to the negative control staining (black lined histogram)

Non-adherent PBMCs were resuspended by pipetting, and adherent cells were harvested using either a 0.05% trypsin solution with EDTA or Accutase (both Gibco, Life technology, Thermo Fisher Scientific) and transferred to 1 ml Micronic tubes (Micronic). Briefly, cells were washed once with cold PBS and resuspended in a final volume of 50 µl antibody mix in cold FACS buffer (PBS supplemented with 1% FCS) containing the antibody in the appropriate titrated concentration for 30 min at 4 °C in the dark. Incubating cells with only staining buffer served as a control. The purity of isolated immune cells was determined using the antibodies CD3 FITC, CD4 PE, CD8 PE, CD14 PE and CD15 FITC. CCR9 expression was investigated separately on all cell types using an antibody against CCR9 (CD199 APC). The PBMCs contained 60% T-lymphocytes (CD3^+^), 46% CD4^+^ T-lymphocytes, 26% CD8^+^ T-lymphocytes and 19% monocytes (CD14^+^). Additionally, the PBMCs exhibited a minor contamination with CD15^+^ granulocytes (2%). The purified CD4^+^ T-lymphocytes were shown to be 86% CD4^+^ with small amounts of CD8^+^ (7%), CD14^+^ (4%) and CD15^+^ (2%) cells. The purity of the isolated CD8^+^ T-lymphocytes was found to be 99%. The surface marker changes on macrophages were analyzed with the antibodies CD14 APCCy7 and CD16 PerCPCy5.5 (general monocyte/macrophage markers), CD80 PE and HLA-DR PECy7 (M1 macrophage polarization markers), and CD206 APC (M2 polarization marker). PBMCs were also stained with HLA-DR PECy7. Additionally, the LIVE/DEAD™ Fixable Aqua Dead Cell Stain Kit (Thermo Fisher Scientific, Waltham, USA) was used to exclude dead cells. All antibodies were purchased from BioLegend, Miltenyi Biotec and Becton Dickinson; dilutions were prepared accordingly to the manufacturers recommendations. After antibody incubation, the samples were washed with cold FACS buffer, resuspended in 1% paraformaldehyde (PFA; Roth, Karlsruhe, Germany) in FACS buffer and transferred to 5 ml FACS tubes (Falcon). Samples were kept at 4 °C in the dark until measurement. Data was acquired using a FACS Canto II device with FACS Diva software (Becton Dickinson, San Jose, CA, USA). Data analysis was performed using FlowJo software (TreeStar Inc., Ashland, OR, USA). Gating strategies for PBMCs and macrophages are shown in Fig. 6.

### Cytokine detection

A LEGENDplex™ human inflammation panel (13-plex) assay (BioLegend, San Diego, USA) was performed according to the manufacturer´s instructions to investigate the secretion of different cytokines (IFN-γ, TNF-α, MCP-1, IL-6, IL-8, IL-10) from the cultivated and polarized macrophages and PBMCs after stimulation. Briefly, lyophilized human inflammation panel standard cocktail was reconstituted with 250 μl assay buffer for 10 min and a standard series from 2.4 pg/ml to 10,000 pg/ml was prepared. The pre-mixed beads bottle was vortexed for 2 min before use. 25 μl of the following reagents were pipetted into each microtube: assay buffer, standard or samples, mixed beads, and detection antibodies. All samples were measured on a FACS Canto II flow cytometer and analyzed using the LEGENDplex™ software (BioLegend, San Diego, USA).

### Chemotaxis assay

Migration was investigated using a modified Boyden-Chamber migration assay. 96-multiwell format ChemoTx plates with polycarbonate membranes (MERCK Millipore, Burlington, USA) were used. The pore sizes were chosen for each cell type individually to enable active migration without cells passing through the pores passively. Diameters of 3 μm were chosen for T-cells, 5 μm for monocytes and PBMCs, and 8 μm for macrophages. Except for the macrophages, which were cultivated, polarized and detached as described previously, all cell types were freshly isolated. A CCL25 dilution series (0.01–1000 nM) in complete cell culture medium without M-CSF was prepared on ice to enable comparison of the chemotactic effect between particles, supernatants and pure CCL25. The buffer of the tested release samples was exchanged as described above. To address the possibility of an inhibitory effect of the release samples on the migration of the immune cell types, the samples were tested undiluted and in 1:10, 1:100 and 1:1000 dilutions. Since a chemotactic reaction of the immune cells could also be attributed to the PLGA fragments in the samples, NL release samples from the same days were tested as a comparison. To quantify the number of migrated cells, an image of each well was taken with the software ProgRes Capture Pro™ (Jenoptik, Jena, Germany). The cell numbers in each picture were determined using the ImageJ plug-in Cell ImageAnalyzer v4.5. The program subtracted the red value from the green value in the RGB color mode, yielding a grey-scale image. The particle analyzer counted the cells using different thresholds for the detection, starting from the adjusted ‘Max-Entropy Threshold’ in *ImageJ*.

### Statistical analysis

*Microsoft Excel* (2011) was used to test datasets for normal distribution using the Lillefors-corrected Kolmogorow-Smirnov-Test. Normally distributed datasets were analyzed using a paired student’s t-test to detect significant changes of receptor expression, cytokine secretion and cell migration of CCL25 stimulated immune cell subsets compared to negative controls. Datasets which were not normally distributed were tested for significant changes with the non-parametric Mann–Whitney-U-Test. Since only one statistical test was performed on every dataset, a Bonferroni-Holms corrected p-value of ^0.05^/_1_ = 0.05 was considered as significant. Due to the low risk of a multiple comparison problem, no additional post-hoc-tests were performed. The *p*-values of the individual analyses are given in the result graphs; results are illustrated with their individually calculated standard deviations.

## Data Availability

The datasets used and analyzed during the current study are available from the corresponding author on reasonable request. In general, data generated or analyzed during this study are included in this published article and its additional information files.
